# Microwave field frequency and current density modulated skyrmion-chain in nanotrack

**DOI:** 10.1038/srep15154

**Published:** 2015-10-15

**Authors:** Fusheng Ma, Motohiko Ezawa, Yan Zhou

**Affiliations:** 1 Temasek Laboratories, National University of Singapore, Singapore; 2 Department of Applied Physics, University of Tokyo, Hongo 7-3-1, Tokyo 113-8656, Japan; 3 York-Nanjing Joint Center for Spintronics and Nano Engineering (YNJC), School of Electronics Science and Engineering, Nanjing University, Nanjing 210093, China; 4 Department of Physics, University of Hong Kong, Hong Kong, P. R. China

## Abstract

Magnetic skyrmions are promising candidates as information carriers for the next-generation spintronic devices because of their small size, facile current-driven motion and topological stability. The controllable nucleation and motion of skyrmions in magnetic nanostructures will be essential in future skyrmionic devices. Here, we present the microwave assisted nucleation and motion of skyrmion-chains in magnetic nanotrack by micromagnetic simulation. A skyrmion-chain is a one-dimensional cluster of equally spaced skyrmions. A skyrmion-chain conveys an integer bit *n* when it consists of *n* skyrmions. A series of skyrmion-chains with various lengths is generated and moved in the nanotrack driven by spin-polarized current. The period, length and spacing of the skyrmion-chains can be dynamically manipulated by controlling either the frequency of the microwave field or the time dependent spin-polarized current density. A skyrmion-chain behaves as a massless particle, where it stops without delay when the current is stopped. Their velocity is found to be linearly dependent on the current density and insensitive to the frequency and amplitude of the excitation microwave field. Uniform motion of trains of skyrmion-chains in nanotrack offers a promising approach for spintronic multi-bit memories containing series of skyrmion-chains to represent data stream.

The dynamical behaviour of magnetic domain walls (DWs) has been extensively investigated recently for their potential application as novel spintronic devices^1^,^2^,^3^,^4^,^5^,^6^,^7^,^8^. The successive nucleation and controlled manipulation of a series of closely spaced DWs in magnetic nanowires by spin-polarized currents via spin-transfer torque mechanism has attracted considerable attention for potential applications as non-volatile magnetic memory devices^1^,^2^,^9^,^10^, logic devices^10^,^11^,^12^, and sensing devices^13^. However, to realize such devices, some challenges must be addressed before this approach can be translated into a competitive technology. Such kinds of challenges are to deal with the difficulty of injecting domain walls into nanowire since it typically requires large localized magnetic field^9^; the large current density required to shift DWs inducing an intensive Joule heating from resistance^14^; the detrimental effects of defects; and the Walker limit of the maximum velocity at which DWs can propagate without structure deformation^15^,^16^,^17^. Other spin textures could offer viable alternatives if their intrinsic properties can help tackle most of the constraints related to DW.

Magnetic skyrmion is a topologically nontrivial particle-like spin texture with a whirling configuration, and it is characterized by a finite topological winding number^18^,^19^,^20^,^21^,^22^,^23^. The discovery of magnetic skyrmion lattice in magnetic materials without spatial inversion symmetry^20^,^21^,^22^,^23^,^24^,^25^,^26^,^27^,^28^ has triggered a flurry of interest in them. Recently, magnetic skyrmions have also been found in magnetic ultrathin films^21^,^23^,^28^, in which the inversion symmetry is broken by the presence of non-equivalent interfaces with Dzyaloshinskii–Moriya interaction (DMI)^29^,^30^. The sizes of the observed magnetic skyrmions are extremely small, ranging from approximately 3 nm to 100 nm depending on material parameters. These novel spin textures are topologically protected and their topological stability drastically reduces the influence of defects so as to avoid a continuous deformation of the field configuration^23^. The weak influence of defects is ascribed to the role of the Magnus force for skyrmions and the advantage of their flexibility to avoid pinning centres^31^. Since the discovery of magnetic skyrmions, numerous efforts were devoted to manipulate their motion allowing for potential applications. It was recently demonstrated experimentally that a spin-polarized current with small current density (~10^6^ A/m^2^) can drive the motion of the skyrmion lattices^32^,^33^, which is about 5 orders of magnitude smaller than that required to shift DWs (~10^11^ A/m^2^)^2^. This has been attributed to their efficient coupling to the current via spin-transfer torque by a spin-Magnus force mechanism^31^,^32^,^34^,^35^. Therefore, benefiting from their topological stability^20^,^36^, nanometric size^36^, and ultralow threshold current density for the motion^32^,^33^, magnetic skyrmions are promising candidate for future spintronic applications, particularly, as information carriers in ultra-dense information memory, logic operation, and other information processing devices^36^,^37^,^38^. Magnetic skyrmion based spintronic devices could be characterized by low cost, high performance, high stability, low power consumption, and non-volatility^37^,^38^.

It is essential to understand how skyrmions can be dynamically nucleated, manipulated, shifted, detected, and annihilated before realizing the proposed device and technology based on their motion. Although most of the reported observations are on the skyrmion lattices in thin films, from the application point of view, skyrmionic devices will require either individual or multiple skyrmions to be efficiently manipulated in magnetic nanostructures. It has been numerically shown that under the influence of spin transfer torques, isolated skyrmions can be created by an electric current in a simple constricted geometry of a notch or by local injection of a spin-polarized current in magnetic nanostructures^37^,^39^,^40^. Recently, the individual writing and deleting single skyrmions using local spin-polarized current from a scanning tunnelling microscope (STM) has also been experimentally demonstrated in an ultrathin magnetic film subjected to an externally applied field^41^. The creation and annihilation of single skyrmions is realized under certain choice of temperature and external magnetic field to prevent thermally activated switching and to tune the energy landscape, respectively. These localized skyrmion with a diameter of a few nanometers in a two atomic layer thick film of palladium and iron on an iridium crystal were imaged by a spin-polarized STM. However, the skyrmion motion of a multi-bit *i.e.* the motion of multiple skyrmions in magnetic nanostructures has barely been presented. The precise and synchronous nucleation and manipulation of multiple skyrmions in magnetic nanotracks will be essential in future skyrmionic device.

Very recently, Zhou and Ezawa have presented a conversion mechanism between a DW pair and a skyrmion by employing nanotracks with different width^42^. Inspired by this conversion mechanism, we demonstrate the sequential injection of multiple magnetic skyrmions and their motion in the nanotrack by micromagnetic simulation. To distinguish them from the reported isolated and sequential skyrmions, we term these equally spaced units of multiple skyrmions as skyrmion-chains. Namely, each skyrmion-chain is a one-dimensional cluster made of equally spaced skyrmions. The microwave field assisted nucleation and motion of skyrmion-chains is driven by spin-polarized current via spin-transfer torques. The static properties of the skyrmion-chains can be manipulated by either controlling the microwave field frequency or changing the spin-polarized current pulse. It is desirable to achieve a train of skyrmion-chains with their properties can be manipulated for potential spintronic applications.

## Results

**Figure 1:**
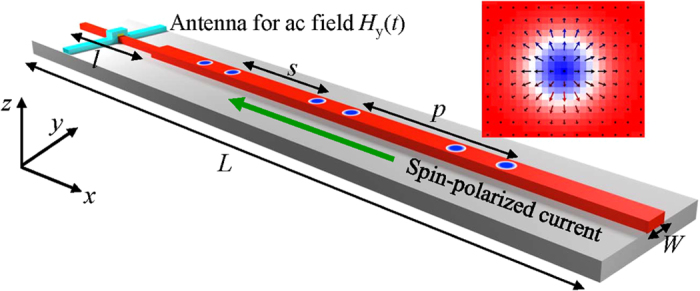
Schematic of the investigated device structure for skyrmion-chain nucleation and motion. The device consists of Co/Pt nanotrack with a narrow end, an antenna for generating microwave field *H*_*y*_(*t*), and the spin-polarized current along the –*x* direction. The inset is the schematic representation of the Néel skyrmion magnetic texture. The color encodes the out of plane component of the magnetization. It changes from the +*z* direction in the center to a complete alignment with the opposite direction in the outer rim. Blue (red) region shows that the *z*-component of the spin is negative (positive), while the white region shows that the spin direction is in-plane. The arrows represent the in-plane component of the magnetization.

We show the schematic representation of the investigated device structure for skyrmion-chain nucleation and motion in Fig. 1. The device consists of Co/Pt nanotrack with a narrow end on the left side, an antenna for generating microwave magnetic field to write DW pairs, and the spin-polarized electron current for shifting DW pairs and skyrmion-chains along the *x*-direction. The total length of the nanotrack *L* is 2400 nm, and the width of the right wide part *W* is 60 nm. To investigate the width effect on the skyrmion-chain motion, the width W is varied from 48 to 140 nm. The length of the narrow end *l* is 200 nm with the width of 20 nm. The inset in Fig. 1 represents the magnetic texture of the Néel skyrmion, where the spin direction forms a radial pattern^22^. The out-of-plane component of the magnetization changes from being fully aligned in the −*z* direction in the centre to a complete alignment along the +*z* direction in the outer rim. The controlled generation of periodic repetitions of skyrmion-chains containing a variable number of skyrmions will be presented. Let us call it *n*-skyrmion-chain if a chain contains *n* skyrmions. We also call *n* the length. The period of the skyrmion-chains is represented by *p*, and the spacing between neighbouring skyrmion-chains denoted by *s* as indicated in Fig. 1. The period *p*, length *n* and spacing *s* of skyrmions in a single skyrmion-chain can be determined by the frequency of the excitation magnetic field and the density of the driving spin-polarized current, respectively.

### Microwave field frequency modulated skyrmion-chains

**Figure 2:**
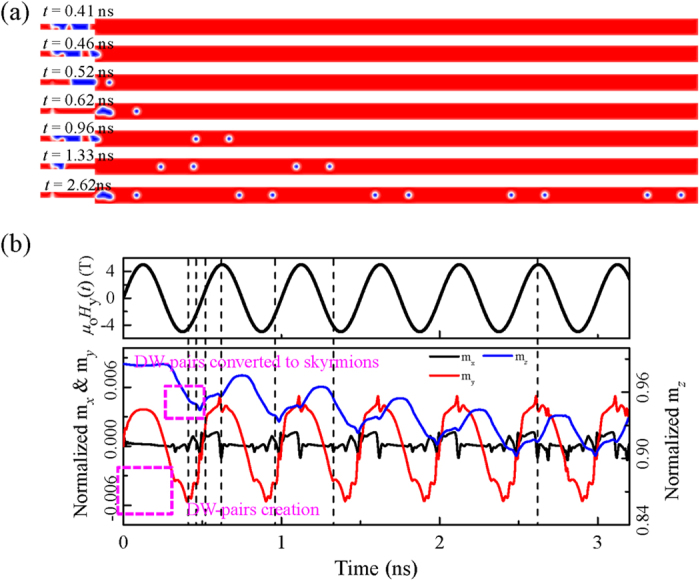
(**a**) Snapshots of a sequential time evolution of the spatial distribution of the local normalized *z* component of the magnetization *m*_*z*_ for the skyrmion-chain motion in the nanotrack at selected time with *j* = 6.25 × 10^13^ A/m^2^, *μ*_o_*H*_o_ = 5 T, and *f* = 2.0 GHz. The magnetization of the nanotrack is pointing to the +*z* direction (red color) and –*z* direction (blue color). (**b**) The time evolution of the phase of the excitation magnetic field *μ*_o_*H*_y_(*t*) and the normalized magnetization *m*_*x*_, *m*_*y*_, *m*_*z*_. The dashed lines indicate the selected times corresponding to the snapshots shown in (**a**).

The nucleation process of the skyrmion-chain of two skyrmions is shown in Fig. 2(a) as serial snapshots of the spatial distribution of the local normalized out-of-plane component of the magnetization *m*_*z*_ with the spin-polarized current density *j* = 6.25 × 10^13^ A/m^2^, the excitation microwave field amplitude *μ*_o_*H*_o_ = 5 T, and the excitation microwave field frequency *f* = 2.0 GHz. The snapshots present a sequential time evolution of the conversion process from DW pairs to skyrmion-chains. The conversion process is similar to that for isolated skyrmions as reported in ref. 42. In contrast to the reported isolated skyrmions, we demonstrate that the number of skyrmions in a single skyrmion-chain, *i.e.* the length of a skyrmion-chain, is controllable. A series of DW pairs are generated by the locally applied microwave magnetic field *H*_y_(*t*) = *H*_o_*sin*(2π*ft*) at a regular time interval in the left narrow end, and then they will propagate rightward along the nanotrack driven by the in-plane injected spin-polarized current. When they reach the junction interface, the DW pairs are continuously deformed into a train of skyrmion-chains. The converted skyrmion-chains are then transmitted continuously in the nanotrack and are destroyed when they reach the right edge of the nanotrack. Hence, trains of skyrmion-chains can be realized by controlled sequential injection of DW pairs. The conversion mechanism can be understood by the fact that the skyrmions can only exist in the nanotrack with its width larger than the diameter of the skyrmions^37^,^40^,^42^. Since the width of the narrow end is smaller than the diameter of skyrmions, the resultant structures are DW pairs. While skyrmions can exist in the wide part of the nanotrack as the width is larger than the diameter of skyrmions. For the real-time dynamical conversion process of *n*-skyrmion-chains with *n* = 1, 2, 3, 4, please refer to the Supplementary Movies 1–4, respectively.

The time evolution of the average normalized magnetization *m*_*x*_, *m*_*y*_, *m*_*z*_are shown in Fig. 2(b). The time-dependent magnetization depends on the presence of DW pairs and skyrmions. The amplitudes of *m*_*x*_ and *m*_*y*_ oscillate periodically around their equilibrium values which is the same as the period of the driving microwave field *H*_y_(*t*). In contrast, the amplitude of *m*_*z*_initially decrease from the equilibrium value as a result of the continuous creation of the skyrmions in the nanotrack as the magnetization of the skyrmion center is opposite to the initial magnetization of the nanotrack. When the first skyrmion reaches the right edge of the nanotrack and annihilates from there, *m*_*z*_ starts to periodically oscillate around the new equilibrium magnetization.

**Figure 3:**
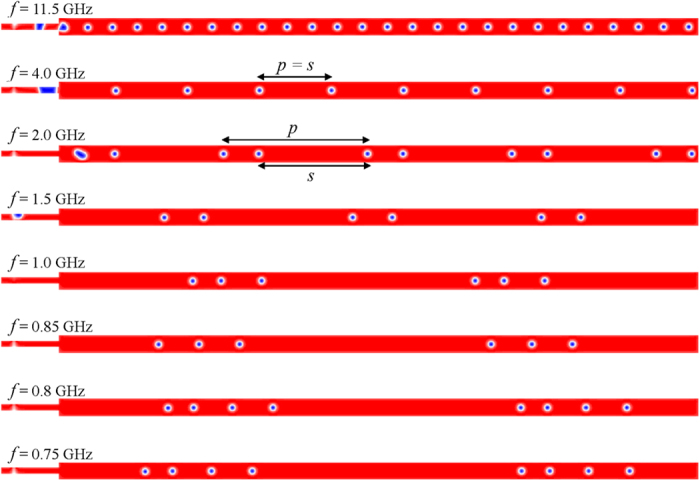
Micromagnetic snapshots of the spatial distribution of the local normalized *z* component of the magnetization for the skyrmion-chain motion in nanotrack under various excitation frequencies *f* with *j* = 6.25 × 10^13^ A/m^2^ and *μ*_o_*H*_o_ = 5 T.

In order to understand the microwave frequency *f* dependence of skyrmion-chain generation process, we investigated the dynamics of the magnetizations in the nanotrack by decreasing the microwave frequency *f* from 11.5 to 0.75 GHz with *j* = 6.25 × 10^13^ A/m^2^, and *μ*_o_*H*_o_ = 5 T. Figure 3 shows the micromagnetic snapshots of the spatial distribution of *m*_*z*_ for skyrmion-chains in nanotrack under various microwave frequencies *f*. It should be pointed out that, for frequencies larger than 11.5 GHz, the DW pairs cannot be converted into skyrmion-chains as they annihilate at the top edge of the narrow end before they reach the narrow-wide junction of the nanotrack (see Supplementary Movie 5). It is observed that the microwave frequency *f* not only affects the length of the skyrmion-chains but also their period. In addition, the spacing between neighboring skyrmion-chains *s* is also affected by the microwave frequency *f*. For instance, there are two skyrmions in a single skyrmion-chain for *f* = 2.0 GHz, while the number is four for *f* = 0.8 GHz. Although both the number of skyrmions in a single skyrmion-chain for *f* = 1.0 and 0.85 GHz is three, the period *p* and the spacing *s* are different. Therefore, the static properties of the skyrmion-chains are highly dependent on the frequency of the excitation microwave field.

**Figure 4:**
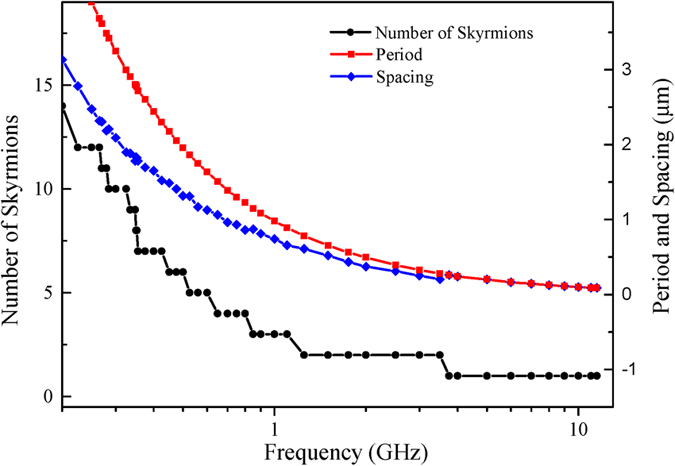
The properties of the skyrmion-chains: the number of skyrmions in single skyrmion-chain, the period of skyrmion-chain, and the spacing between neighbouring skyrmion-chains as a function of the excitation frequencies *f* with *j* = 6.25 × 10^13^ A/m^2^ and *μ*_o_*H*_o_ = 5 T.

The dependence of the length, period and spacing of the skyrmion-chains on the excitation frequencies *f* with *j* = 6.25 × 10^13^ A/m^2^ and *μ*_o_*H*_o_ = 5 T is summarized and plotted in Fig. 4. It is observed that the length of the skyrmion-chains is stepped up from 1 to 13 when the *f* decreases from 11.5 to 0.2 GHz. In contrast to the stepwise increasing of the length of skyrmion-chains, the period *p* and the spacing *s* continuously increase with decreasing the frequency *f* from 11.5 to 0.2 GHz, and they are inversely proportional to the frequency *f*. As illustrated in Fig. 2(b), the DW-pairs are only nucleated in the second half period of the microwave field *H*_y_(*t*) = *H*_o_*sin*(2π*ft*) from (2*N* + 1)π/*f* to (2*N* + 2)π/*f* with *N* being the integer. By changing the frequency *ω*_H_, the period of the microwave field is changed resulting in the variation of the time interval for DW-pairs creation. Therefore, the length of the skyrmion-chains is highly dependent on the microwave field frequency *f*.

### Spin-polarized current density modulated skyrmion-chains

**Figure 5:**
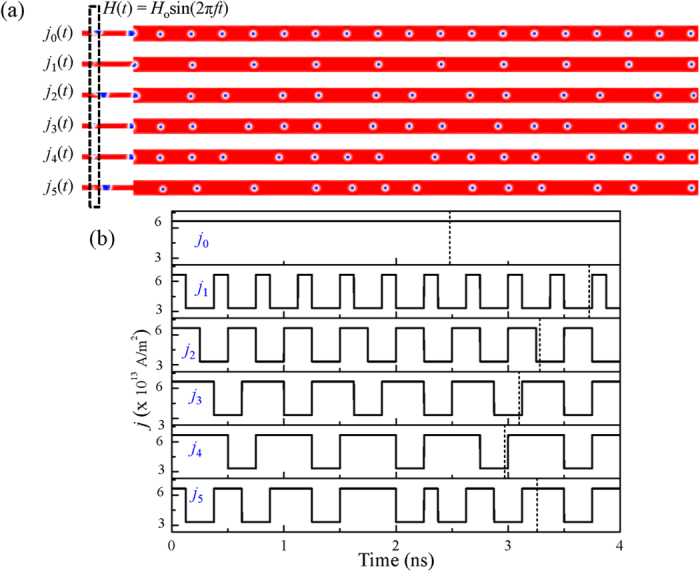
Dynamically controlled nucleation and motion of various skyrmion-chains by manipulating the intensity of the spin-polarized current. (**a**) The snapshots of the magnetization configuration for skyrmion-chains under various current pulses at selected times corresponding to the vertical lines in (**b**) with *μ*_o_*H*_o_ = 5 T, and *f* = 8.0 GHz. (**b**) The intensity of pulsed current density *j* as a function of time. The dashed lines indicate the selected times corresponding to the snapshots shown in (**a**).

We have demonstrated that it is possible to control the properties of skyrmion-chains by changing the microwave field frequency *f* in a single nanotrack. However, in practical applications, we need to use multiple nanotrack arrays for ultra-dense applications. We show an array of six nanotracks as shown in Fig. 5(a). If we use the frequency modulated microwave to realize skyrmion-chains independently each nanotrack needs a sole antenna to generate microwave field of specific frequency, which obviously hinders the integration of ultra-dense devices. Preparing microwave emitting antenna with different frequencies to change the properties of the skyrmion-chains in each nanotrack of the arrays is not convenient. Alternatively, the properties of the skyrmion-chains can be dynamically controlled by manipulating the intensity of the spin-polarized current without changing the frequency of the microwave field. As shown in Fig. 5(a), only one antenna is applied to the array of six nanotracks, then the creation of DW pairs in them are synchronous. By injecting a series of multiple pulsed currents into each nanotrack, the properties of the skyrmion-chains can be controlled by the profile of the current *j*(*t*). The density of the pulsed current *j*_0_(*t*) − *j*_5_(*t*) are periodically manipulated between 6.25 × 10^13^ and 3.0 × 10^13^ A/m^2^. As the DW pairs can only be moved in the nanotrack when *j* is above the threshold value ~5.0 × 10^13^ A/m^2^. Hence, the DW pairs created in the duration *j*(*t*) = 6.25 × 10^13^ A/m^2^ can be transferred and converted into skyrmions, while those created in the duration *j*(*t*) = 3.0 × 10^13^ A/m^2^annihilate at the edge of the nanotrack and cannot be converted into skyrmions. To investigate the effect of *j*(*t*), we record *j*(*t*) as series of time intervals ∆*t* = ∆*t*_*up*_ + ∆*t*_*down*_ with the value of *j*(*t*) of either 6.0 × 10^13^ A/m^2^ or 3.0 × 10^13^ A/m^2^ in ∆*t*_*up*_ and ∆*t*_*down*_, respectively. The current density *j*(*t*) will be recorded as *j*(1, 0) if ∆*t*_*up*_ = 1 ns and ∆*t*_*down*_ = 0 ns. As shown in Fig. 5, for a constant current *j*_0_(1, 0), a train of isolated skyrmions, *i.e.* 1-skyrmion-chains, are generated. However, for the pulse current *j*_1_(0.1, 0.2), the 1-skyrmion-chains are generated, but the spacing between neighbouring skyrmions are enlarged. Similarly, the 2-skyrmion-chains can be generated by injecting pulsed current in the form of *j*_2_(0.25, 0.25). The 3-skyrmion-chains can be generated with *j*_3_(0.4, 0.1). The 4-skyrmion-chains can be generated with *j*_4_(0.5, 0.2). Additionally, a train of multi-skyrmion-chains can also be generated according to how the current density *j*(*t*) is coded, *i.e., j*_5_(*t*) in Fig. 5. (The dynamical process of the train of multi-skyrmion-chains is shown in Supplementary Movie 6) Hence, a train of skyrmion-chains in the form of a combination of arbitrary numbers, can be easily realized according to the requirement by varying the density of the current.

### Velocity of skyrmion-chains and its dependence

**Figure 6:**
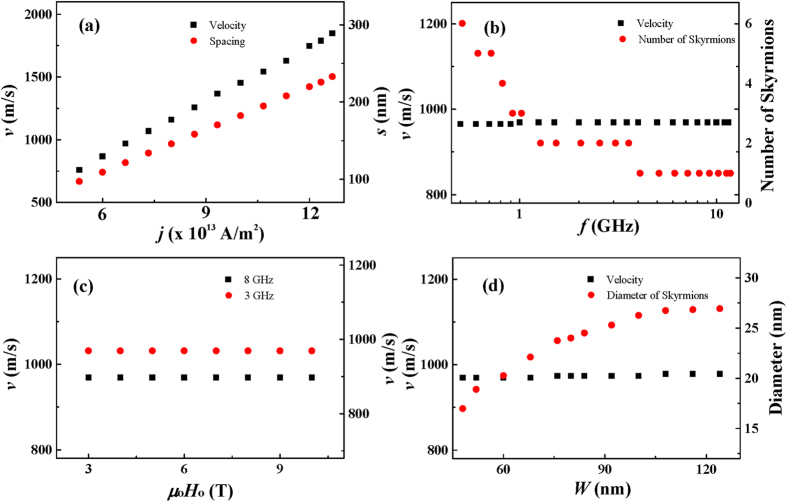
Skyrmion-chain velocity *v* as a function of: (**a**) current density *j* with *f* = 8 GHz, *μ*_o_*H*_o_ = 5 T, and *W* = 60 nm; (**b**) excitation frequency *f* with *j* = 6.25 × 10^13^ A/m^2^, *μ*_o_*H*_o_ = 5 T, and *W* = 60 nm; (**c**) amplitude of the excitation field *μ*_o_*H*_o_ with *j* = 6.25 × 10^13^ A/m^2^, *f* = 8 GHz, and *W* = 60 nm; and (**d**) width of nanotrack *W* with *j* = 6.25 × 10^13^ A/m^2^, *f* = 8 GHz, and *μ*_o_*H*_o_ = 5 T.

To use magnetic skyrmions as information carrier in the spintronic memory and logic devices, the dynamical properties of the skyrmion is intriguing. Fig. 6 shows the dependence of the skyrmion-chain velocity on the spin-polarized current intensity *j*, the excitation microwave field frequency *f*, the excitation microwave field amplitude *μ*_o_*H*_o_, and the width of the nanotrack *W*. The velocity of the skyrmion-chain as a function of the spin-polarized current density *j* is shown in Fig. 6a. The results show that the velocity of skyrmion-chains, irrespective of the number of skyrmions in each skyrmion-chain, is linearly dependent on the current density *j*. The velocity increased linearly from 760 to 1850 m/s when *j* increases from 5.0 × 10^13^ to 12.0 × 10^13^ A/m^2^. For the current density below ~5.0 × 10^13^ A/m^2^ and above ~12.0 × 10^13^ A/m^2^, the DW pairs collapse and the spin texture is attached to one edge of the nanotrack and is elongated to form a meron^43^,^44^. The motion of skyrmion-chains of different numbers of skyrmions is also simulated, and there is no significant difference in the velocities obtained for different intensities of applied current. The results illustrate that the velocity of the skyrmion-chains is in proportion to the applied current density and independent of the number of skyrmions in each skyrmion-chain. The velocity of the skyrmion-chains as a function of the microwave field frequency *f* and amplitude *μ*_o_*H*_o_ as well as the nanotrack width *W* are shown in Fig. 6b–d. The results show that the velocity is almost independent of the *f, μ*_o_*H*_o_ and *W*. However, the number of skyrmions in one single skyrmion-chain is highly dependent on the frequency *f*. In addition, *μ*_o_*H*_o_ has a strong impact on the creation of DW pairs in the narrow end of the nanotrack. The critical amplitude is 2 T, above which the DW pairs can be continuously generated. It is also observed that the diameter of the skyrmions depends on the nanotrack width *W*. The diameter increases from 17 to 27 nm when the width of the nanotrack increases from 48 to 140 nm. For the width of the nanotrack below 48 nm, the DW pairs cannot be converted into skyrmion-chains.

## Discussion

**Figure 7:**
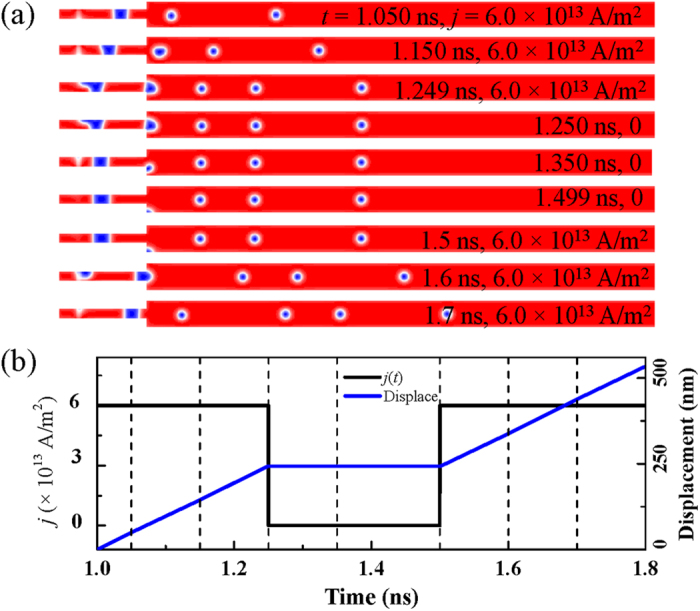
Current density modulated skyrmion-chains in naotrack. (**a**) The snapshots of the magnetization configuration for skyrmion-chains at selected times corresponding to the vertical dashed lines in (**b**) with *μ*_o_*H*_o_ = 5 T, and *f* = 8.0 GHz. (**b**) The current density *j* as a function of time in a time interval and the corresponding skyrmion-chain displacement. The dashed lines indicate the selected times corresponding to the snapshots shown in (**a**).

A skyrmion-chain behaves like a massless particle without changing its structure. They maintain their spin structures and the initial spacing between skyrmions remain almost a constant while they are moving uniformly in the electron flow direction. The response of the multi-skyrmion-chains to multi-pulsed current *j*_5_(*t*) is shown in Fig. 7 for the time interval from 1.0 to 1.8 ns. The skyrmion-chains are moved uniformly with a velocity of 195 m/s from 1.0 to 1.25 ns. They immediately stop without any delay when the density of the current is reduced to 0. In the time interval from 1.25 to 1.5 ns, as the current density is 0, the skyrmion-chains are stationary at the position where the current is switched off. Once the current is switched on at 1.5 ns, the skyrmion-chains start to move at the velocity of 195 m/s. These massless dynamic characteristics show that the skyrmion-chains behave as if they were independent particles. This is because that the skyrmion-skyrmion repulsion is short-ranged and they do not interact when they are seperated^45^.

The static properties of the proposed skyrmion-chain are quite related to the frequency of the excitation magnetic field *f*, the amplitude of the excitation magnetic field *μ*_o_*H*_o_, and the density of the driven spin-polarized current *j*. Depending on the requirement, suitable values of the three parameters can be chosen. A relatively high amplitude of the excitation magnetic field *μ*_o_*H*_o_ = 5 T is used for the results presented here, similar results can also be reproduced at smaller excitation field, such as *μ*_o_*H*_o_ = 1.5 T (see Supplementary Figure 1). Actually, it has been demonstrated that the conversion between domain wall pair and skyrmion remains very robust against a wide range of the saturation magnetization *M*_s_ (*i.e.*, 0 < *M*_s_ < 8.8 × 10^5^ A/m) and a wide range of the perpendicular magnetic anisotropy *K* (*i.e.*, 0.4 MJ/m^3^ ≤ *K* ≤ 0.9 MJ/m^3^)^42^. Similarly, the proposed conversion between domain wall pairs and skyrmion-chains also remains very robust against a wide range of *M*_s_ and *K*. We have carried out a series of simulations by reducing *K* from 0.7 MJ/m^3^ to 0.4 MJ/m^3^ with all the other material parameters unchanged. The skyrmion-chains with different length *n* are converted from the domain wall pairs (see Supplementary Figure 2). The amplitude of the excitation magnetic field *μ*_o_*H*_o_ is only 0.3 T, and the density of the driven spin-polarized current *J* is decreased to 6.0 × 10^12^ A/m^2^, which is one order of magnitude smaller than that used in Fig. 2.

To investigate the effect from the non-adiabatic spin torques, different values of the non-adiabatic parameter *β* (keeping the Gilbert damping *α* = 0.3). As shown in Supplementary Figures 3 and 4, for *β* = *α* = 0.3, there is no transverse motion, and the skyrmion-chains move longitudinally along the central line of the track without transverse motions. For *β* = 0.28, the converted skyrmion-chain has a longitudinal velocity in the *x*-direction and also a transverse velocity in the –*y*-direction (see Supplementary Figure 3b). For *β* = 0.35, the converted skyrmion-chain has a longitudinal velocity in the *x*-direction and also a transverse velocity in the +*y*-direction (see Supplementary Figure 3d). The transverse motion along *y* stops at some distance from the edge because of the repulsive interaction due to the tilting of magnetization on the track edges induced by DMI^40^,^42^. However, the domain-wall pairs cannot convert to skyrmions when *β* is too different from *α* (see Supplementary Figure 3a,3e). Hence, even when *β* ≠ *α*, the presented conversion mechanism from domain-wall pairs to skyrmion is also obtained provided *β* is not too different from *α*. The converted skyrmion-chains can still travel along the nanowire even when *β* is not exactly equal to *α* due to the skyrmion-edge repulsive effect. Additionally, the skyrmion-chains still behaves as massless particles, where they stop without delay when the current is stopped (see Supplementary Figure 5).

We have presented the skyrmion-chain motion in the nanotrack with perfect structures. There will be unavoidable defects for nanoscale devices limited by the lithography process. Similar to the reported topologically protected stability of isolated skyrmions, the presented skyrmion-chains are also topologically protected with their uniformity unchanged by tiny defect in the track. As shown in Supplementary Figures 6 and 7, the notches will not have significant effects on both the static and dynamic properties of the generated skyrmion-chains. The skyrmion-chains can pass the notches if the size of the notches is not too large. However, for larger notch of ~50 nm, the skyrmion will touch the triangular edges and then be destroyed.

We have investigated the current-induced dynamics of a train of skyrmion-chains in a nanotrack. We find that the frequency of the exciting microwave field drastically change the static properties of the skyrmion-chains, *i.e.* the number of skyrmions in a single skyrmion-chain, the period of skyrmion-chains, and the spacing between neighbouring skyrmion-chains. Furthermore, we also demonstrated that the properties of the skyrmion-chains are controllable by dynamically manipulating the density of the current. The proposed skyrmion-chains are characteristic of massless mobility, independent of the number of skyrmions in them. They maintain their spin structures and keep the initial spacing between skyrmions when they are moving uniformly in the nanotrack as if they were independent skyrmions. The velocity of the skyrmion-chains is observed to be linearly dependent on the spin-polarized current density and shows a weak dependence on the excitation microwave frequency and field amplitude. We have proposed to use a skyrmion-chain as an information carrier, where the bit *n* is assigned for the *n*-skyrmion-chain. Our findings could provide a strategy for the design of skyrmion-based racetrack memories and logic devices, which are appealing from the perspective of using trains of skyrmion-chains in nanotracks for dense encoding of information.

## Methods

### Micromagnetic simulations

We investigate the dynamical properties of such skyrmion-chains with micromagnetic simulations as well as theoretical analysis. The simulations are performed with the public object-oriented micromagnetic framework (OOMMF) code^46^ which was extended to consider the Dzyaloshinskii-Moriya interaction^47^,^48^,^49^ and the current-induced magnetization dynamics as described by the Landau-Lifshitz-Gilbert equation with additional spin-transfer torque terms^50^,^51^:
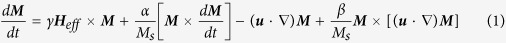
where ***M*** is the local magnetization, 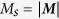 the saturation magnetization, *γ* the gyromagnetic ratio, ***H***_*eff*_ the effective field, *α* the Gilbert damping factor, and *β* the nonadiabatic spin-transfer parameter^50^,^51^.

The local effective magnetic field ***H***_*eff*_ includes the exchange, anisotropy, magnetostatic, and Dzyaloshinskii-Moriya fields. The Dzyaloshinskii-Moriya field caused by the interfacial DMI is given in a continuous form^47^,^48^:
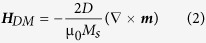
where *D* is the continuous effective DMI constant in mJ/m^2^, *μ*_0_ is the magnetic permeability, and *M*_s_ is the saturation magnetization.

The vector ***u*** representing the spin-polarized current density (the spin drift velocity, in m/s), is defined as
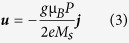
where ***j*** is the current density, *g* is the Landé factor, *μ*_*B*_ the Bohr magnetron, *e* the electron charge and *P* the polarization rate of the current. Electrons flowing toward the right, *i.e.* the current flows toward the left mean that *u *> 0. The skyrmion should move in the direction of propagation of the electrons.

Material parameters of the nanotracks used in the simulations are those of cobalt (Co) on a platinum (Pt) substrate inducing DMI as follows^40^: the saturation magnetization *M*_s_ = 5.8 × 10^5^ A/m, the exchange stiffness *A* = 1.5 × 10^−11^ J/m, and perpendicular magnetic anisotropy *K* = 0.7 MJ/m^3^, the DMI constant *D* = 3 mJ/m^2^, the damping constant *α* = 0.3, the nonadiabatic spin-transfer parameter *β* = 0.3, the gyromagnetic ratio *γ* = 2.211 × 10^5^ m/As, and the polarization rate of the current *P* = 0.4. The cell size used in the simulation is 1 × 1 × 1 nm^3^, which is well below the characteristic domain wall length. All simulations are performed without the application of external magnetic field. For the material parameters of Co used in the simulation, *g* = 2, *u* ≈ j*P*(4 × 10^−11^ m^3^/As). If *u* = 1000 m/s, the current density is *j* ≈ 6.25 × 10^13^ A/m^2^.

For the successive generation of series of DW pairs, an external harmonic sinusoidal microwave magnetic field *H*_*y*_(*t*) = *H*_o_*sin*(2π*ft*) with the field frequency *f* and field amplitude *μ*_o_*H*_o_ is applied locally to a Δ*x* × Δ*y* × Δ*z* = 10 × 20 × 1 nm^3^ section in the left narrow end of the nanotrack by means of injection of ac current pulses. The influence of the field distribution Δ*x* is simulated as shown in the Supplementary Figure 8. It is found that similar results are achieved for Δ*x* above 6 nm. But for Δ*x* below 6 nm, the microwave field does not generate domain wall pairs and hence without the skyrmion generation. With the effect of this microwave field, the direction of the magnetization can be locally reversed and then two DWs are nucleated. Additionally, to shift the generated DW pairs rightward along the nanotrack, a spin-polarized current is injected in-plane in the negative *x*-direction where electrons flow in positive *x*-direction. The DW pairs are moved and converted into skyrmions when they pass through the narrow-wide junction part of the nanotrack.

The micromagnetic simulation model used in this work neglects the modification of the current electrons by the skyrmion, such as the generation of current inhomogeneities due to the scattering of electrons on the magnetization gradients. However, the modification of the current electrons by the skyrmion will be tiny, and this effect will not be significant for the present work. In order to investigate the modification of the current electrons by the skyrmions, a self-consistent model is recently proposed by coupling the Landau-Lifshitz equation through the spin-transfer torque term with the Schrödinger equation for the itinerant spins where electrons obey quantum dynamics^52^,^53^. In the self-consistent model, the strong inhomogeneities are spontaneously created by the interaction of the electrons and the skyrmions. The torque exerted by the itinerant spins modifies the distribution of the magnetization around the skyrmion core and tends initially to reduce its size and ultimately drives a topological change.

## Additional Information

**How to cite this article**: Ma, F. *et al.* Microwave field frequency and current density modulated skyrmion-chain in nanotrack. *Sci. Rep.* **5**, 15154; doi: 10.1038/srep15154 (2015).

## Supplementary Material

Supplementary Information

Supplementary Movies 1

Supplementary Movies 2

Supplementary Movies 3

Supplementary Movies 4

Supplementary Movies 5

Supplementary Movies 6
