# Supplementary material for: Microwave field frequency and current density modulated skyrmion-chain in nanotrack
Source: Sci Rep. 2015 Oct 15;5:15154. doi: 10.1038/srep15154 (PMC4606831; doi:10.1038/srep15154)
Supplement: Supplementary Information [file srep15154-s1.pdf]

Supplementary Information for:

Microwave field frequency and current density modulated skyrmion-chain in nanotrack

Fusheng Ma[1], Motohiko Ezawa[2], & Yan Zhou[3, 4]

[1]Temasek Laboratories, National University of Singapore, Singapore, [2]Department of Applied Physics, University of Tokyo, Hongo 7-3-1, Tokyo 113-8656, Japan, [3]York-Nanjing Joint Center for Spintronics and Nano Engineering (YNJC), School of Electronics Science and Engineering, Nanjing University, Nanjing 210093, China, [4]Department of Physics, University of Hong Kong, Hong Kong, P. R. China.

I. Supplementary Movies

Supplementary Movie 1. Real-time dynamical conversion process of 1-skyrmion-chain.

Supplementary Movie 2. Real-time dynamical conversion process of 2-skyrmion-chain.

Supplementary Movie 3. Real-time dynamical conversion process of 3-skyrmion-chain.

Supplementary Movie 4. Real-time dynamical conversion process of 4-skyrmion-chain.

Supplementary Movie 5. For the frequency of microwave field larger than 11.5 GHz, the created domain-wall pairs cannot be converted into skyrmion-chains.

Supplementary Movie 6. Real-time dynamical conversion process of the multiple skyrmion-chain: multi-skyrmion-chain.

II. Supplementary Figures

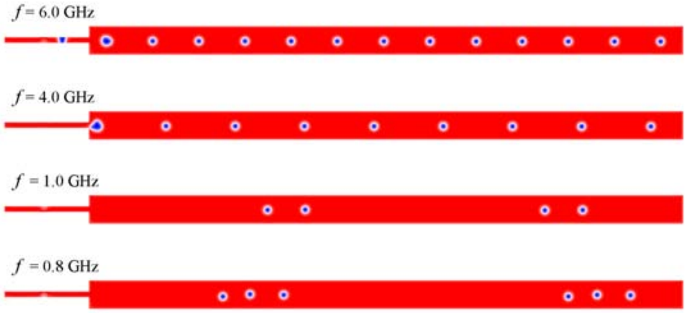

Supplementary Figure 1. Micromagnetic snapshot images of the spatial distribution of the local normalized z component of the magnetization for the skyrmion-chain motion in nanotrack under various excitation frequencies f with $j = 6.25 \times 10^{13}$ A/m^2 and $\mu_o H_o = 1.5$ T.

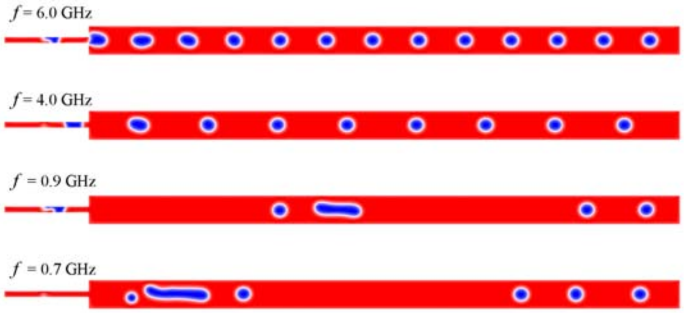

Supplementary Figure 2. Micromagnetic snapshot images of the spatial distribution of the local normalized z component of the magnetization for the skyrmion-chain motion in nanotrack under various excitation frequencies f with $j = 6.0 \times 10^{12}$ A/m^2 and $\mu_o H_o = 0.3$ T. The perpendicular magnetic anisotropy K is reduced from 0.7 MJ/m^3 to 0.4 MJ/m^3 with all the other material parameters the same as those used in Supplementary Figure 1. The skyrmion-chains with different length n are converted from the domain wall pairs. The $\mu_o H_o$ is only 0.3 T, and the J is of the value 6.0×10^{12} A/m^2, which is one order of magnitude smaller than that used in Supplementary Figure 1.

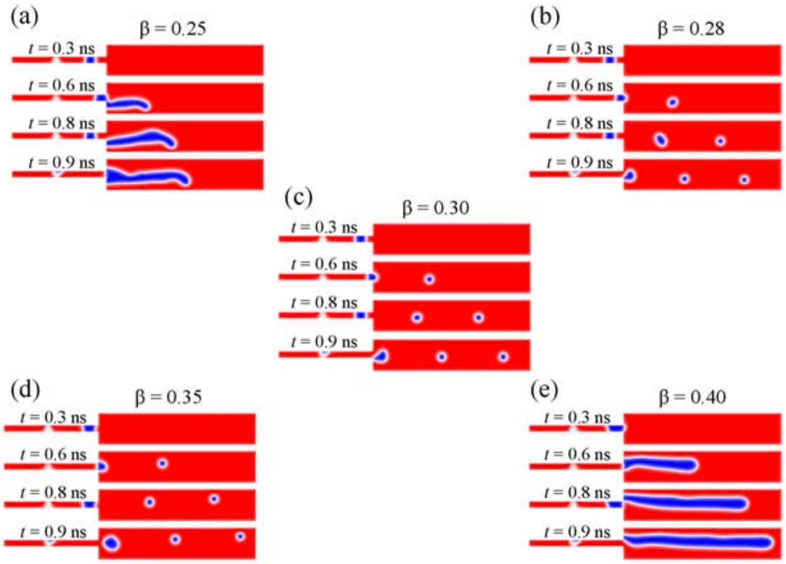

Supplementary Figure 3. Effect of non-adiabatic STT coefficient β. Micromagnetic snapshot images of the spatial distribution of the local normalized z component of the magnetization for the skyrmion-chain motion in nanotrack with j = 5.0 × 10^{13} A/m^2, $\mu_o H_o$ = 5 T, and f = 4.0 GHz. (a) β = 0.25; (b) β = 0.28; (c) β = 0.30; (d) β = 0.35; (e) β = 0.40. For all the cases, damping coefficient α = 0.3.

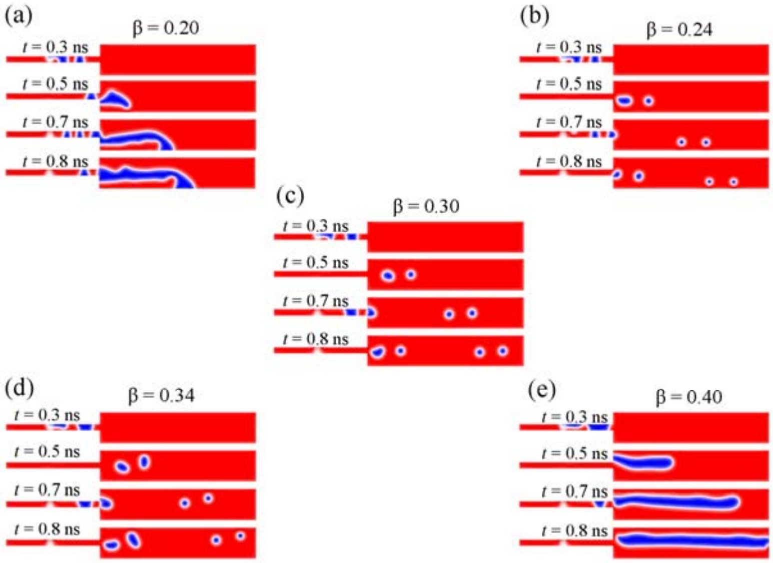

Supplementary Figure 4. Effect of non-adiabatic STT coefficient β. Micromagnetic snapshot images of the spatial distribution of the local normalized z component of the magnetization for the skyrmion-chain motion in nanotrack with j = 6.25 × 10^{13} A/m^2, $\mu_o H_o$ = 5 T, and f = 3.0 GHz. (a) β = 0.20; (b) β = 0.24; (c) β = 0.30; (d) β = 0.34; (e) β = 0.40. For all the cases, damping coefficient α = 0.3.

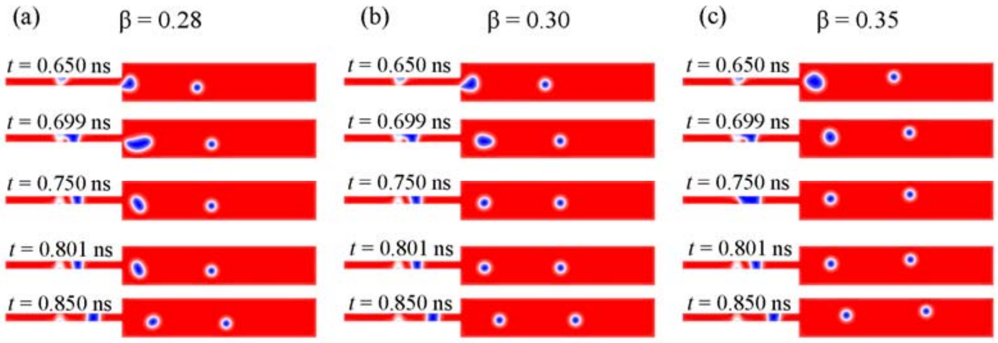

Supplementary Figure 5. Effect of non-adiabatic STT coefficient β. Micromagnetic snapshot images of the spatial distribution of the local normalized z component of the magnetization for the skyrmion-chain motion in nanotrack with j = 5.0 × 10^{13} A/m^2, $\mu_o H_o$ = 5 T, and f = 4.0 GHz. (a) β = 0.20; (b) β = 0.30; (c) β = 0.35. For all the cases, damping coefficient α = 0.3. The current is switched off at t = 0.7 ns, and then switched on at t = 0.8 ns.

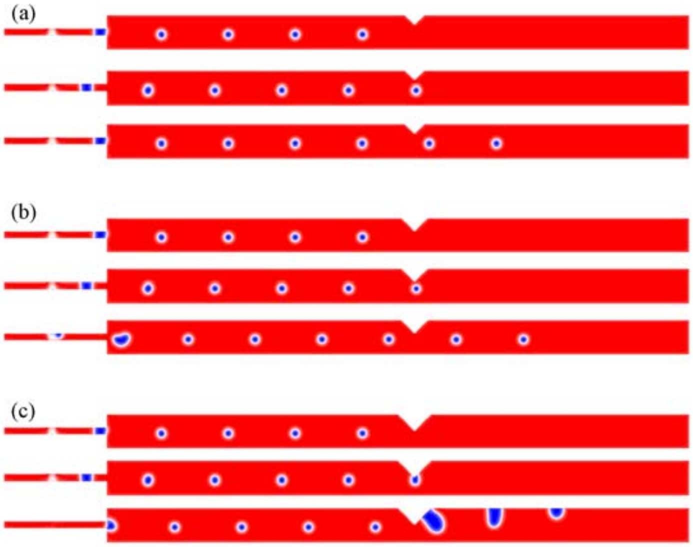

Supplementary Figure 6. Snapshots of a sequential time evolution of the spatial distribution of the local normalized z component of the magnetization m_z for the skyrmion-chain motion in the nanotrack with defect at selected times with j = 5.0 × 10^{13} A/m^2, $\mu_o H_o$ = 5 T, and f = 4.0 GHz. A physical notch is placed on the wide part of the track. The notch size is around (a) 30 nm, (b) 40 nm, and (c) 50 nm. The width of the narrow and the wide part of the nanotrack are 20 nm and 100 nm, respectively. The total length of the nanowire is 2000nm.

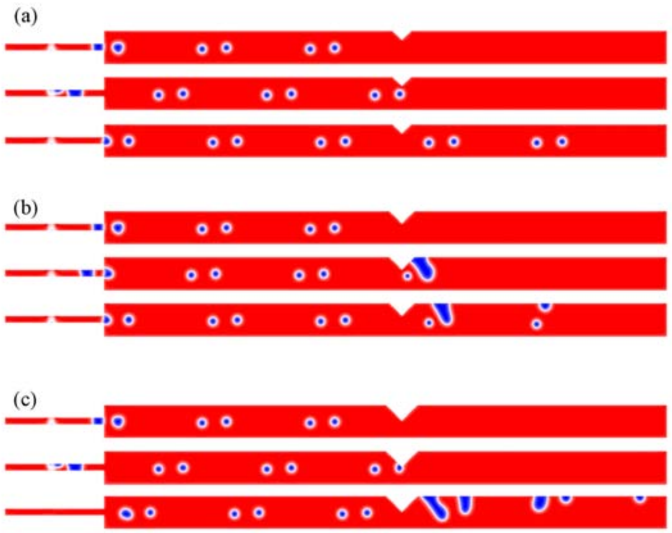

Supplementary Figure 7. Snapshots of a sequential time evolution of the spatial distribution of the local normalized z component of the magnetization m_z for the skyrmion-chain motion in the nanotrack with defect at selected times with $j = 6.25 \times 10^{13}$ A/m^2, $\mu_o H_o = 5$ T, and $f = 3.0$ GHz. A physical notch is placed on the wide part of the track. The notch size is around (a) 30 nm, (b) 40 nm, and (c) 50 nm. The width of the narrow and the wide part of the nanotrack are 20 nm and 100 nm, respectively. The total length of the nanowire is 2000nm.

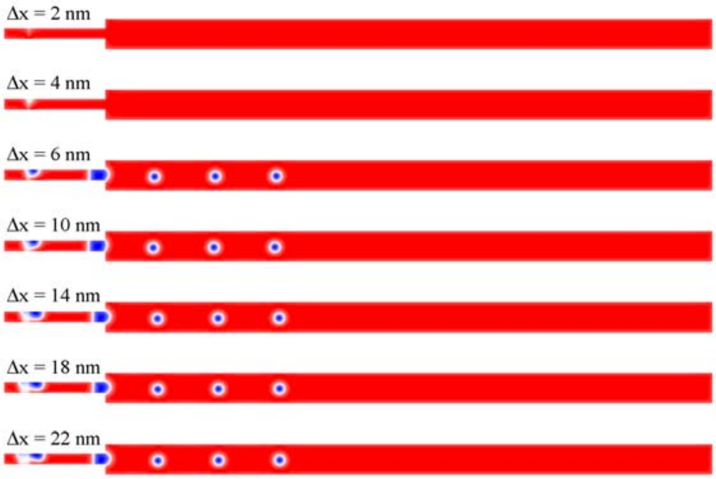

Supplementary Figure 8. Micromagnetic snapshot images of the spatial distribution of the local normalized z component of the magnetization for the skyrmion-chain motion in nanotrack for various Δx with $f = 8$ GHz, $j = 6.25 \times 10^{13}$ A/m^{2}and $\mu_o H_o = 5$ T. Δx is the region, where the excitation microwave field $H_y(t)$ is applied to.
