# Supplementary figures and images for: Microwave field frequency and current density modulated skyrmion-chain in nanotrack
Source: Sci Rep. 2015 Oct 15;5:15154. doi: 10.1038/srep15154 (PMC4606831; doi:10.1038/srep15154)

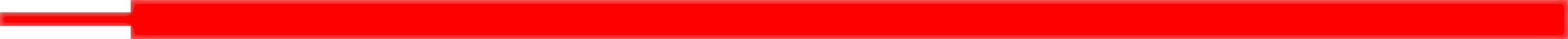

Supplement: Supplementary Movies 1 [file srep15154-s2.gif]

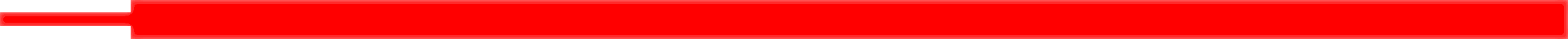

Supplement: Supplementary Movies 2 [file srep15154-s3.gif]

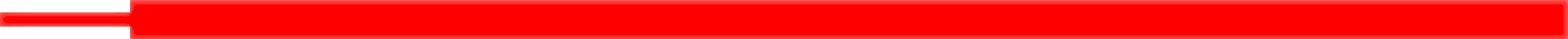

Supplement: Supplementary Movies 3 [file srep15154-s4.gif]

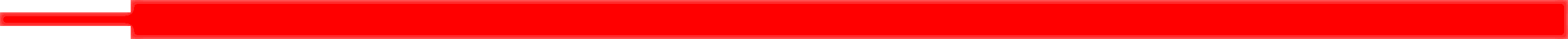

Supplement: Supplementary Movies 4 [file srep15154-s5.gif]

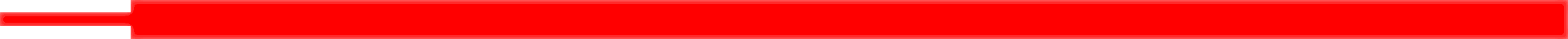

Supplement: Supplementary Movies 5 [file srep15154-s6.gif]

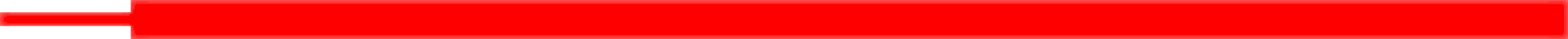

Supplement: Supplementary Movies 6 [file srep15154-s7.gif]
